# Development and validation of the Sorting non-trauMatIc adoLescent knEe pain (SMILE) tool – a development and initial validation study

**DOI:** 10.1186/s12969-021-00591-5

**Published:** 2021-07-06

**Authors:** Clara Guldhammer, Sinead Holden, Marina Elmelund Sørensen, Jens Lykkegaard Olesen, Martin Bach Jensen, Michael Skovdal Rathleff

**Affiliations:** 1 Center for General Practice at Aalborg University, Fyrkildevej 7, 9220 Aalborg, Denmark; 2 Department of Health Science and Technology, Faculty of Medicine, Aalborg University, Aalborg, Denmark

**Keywords:** Adolescents, Knee pain, General practice, Sports medicine

## Abstract

**Background:**

Despite the commonality of adolescent knee pain, there are no tools to support medical doctors to correctly diagnose knee pain. This study aimed to develop and evaluate a support tool for diagnosing the most common types of non-traumatic adolescent knee pain.

**Method:**

A systematic search on Medline identified the literature on clinical tests and diagnoses of adolescent knee pain. The search was supplemented by textbooks and transformed into a diagnostic flowchart based on onset, symptoms, and pain localisation. This tool was revised based on feedback from general practitioners and experts in sports medicine. The tool was evaluated on two separate days with blinded assessors. Overall, 27 participants (aged 10–17 years) with non-traumatic knee pain were included. All participants were diagnosed by medical doctors or medical students, without and with the use of the tool. Diagnoses were compared to a gold standard (expert clinician). An interview to inform optimisations of the tool was performed with the assessors. Percentage agreement with the gold standard, and Kappa statistic for interrater reliability were calculated.

**Results:**

The final tool improved diagnostic agreement with the gold standard from 22.7% (95% CI 10.3–35.1) to 77.3% (95% CI 64.9–89.7). Inter-rater reliability increased from *poor* agreement k = − 0.04 (95% CI, − 0.12-0.04*)* to *moderate* agreement k = 0.56 (95% CI, 0.40–0.72).

**Conclusion:**

This simple diagnostic tool is quick to use and may assist doctors in diagnosing non-traumatic knee pain in adolescents.

**Supplementary Information:**

The online version contains supplementary material available at 10.1186/s12969-021-00591-5.

## Background

Annually, 7% of adolescents visit their general practitioner with musculoskeletal problems [1]. Musculoskeletal problems are one of the most common causes of consultation in this population [1]. Knee pain is common among adolescents [2] and often due to non-traumatic causes [3, 4]. Non-traumatic knee pain is most often the result of repetitive and excessive stress to musculoskeletal structures from sports participation and physical activity [5]. ‘The most common types of non-traumatic knee pain during adolescence are Osgood-Schlatter disease and patellofemoral pain [6, 7]’. Despite being less common, Sinding-Larsen Johansson/patellar tendinopathy, Iliotibial band syndrome, and plica syndrome are important differential diagnoses for adolescents with non-traumatic knee pain [5]. In addition to the non-traumatic knee complaints, knee pain can be related to growth also known as benign nocturnal pains of childhood (growing pain), referred pain from the hip or lumbar spine, osteosarcomas, and systemic causes which all are important differential diagnoses [5, 8].

Non-traumatic knee complaints can be long-lasting, with an impact on health-related quality of life and physical activity [9–11]. In light of this, the management strategy initiated by the health care practitioner becomes critical to improve long term prognosis. Furthermore, treatment may need to be managed differently related to the specific diagnosis [12] which can be a challenge for medical doctors, due to non-specific symptoms and clinical tests being of limited diagnostic value [2, 13]. Further, qualitative research shows the importance to adolescents of “getting a name” for their knee pain which underlines the need to support the health care practitioner in diagnosing the specific type of knee pain [14]. The aim of this study was to develop and evaluate a support tool for diagnosing the most common types of non-traumatic adolescent knee pain.

## Methods

### Study design and setting

This study consists of the development and evaluation of the Sorting non-trauMatIc adoLescent knEe (SMILE) tool for non-traumatic knee pain in adolescents. The study was conducted at the Center for General Practice at Aalborg University between February 2019 – February 2020 and inspired by requests from our reference group of general practitioners (GPs).

### Step one: development of the SMILE tool

#### Systematic search

The systematic literature search was conducted in Medline (via PubMed). The search strategy was developed using medical subject headings and text words related to knee, pain, and diagnosis. The search strategy is available in Additional file 1. No language restriction was applied to the search. We included papers published between 1950 and until 1st of March 2019. We also conducted a hand search including the reference lists of included studies and the authors’ personal files to make sure that all relevant material has been captured.

Eligibility criteria for including articles (both narrative and systematic) were articles describing any type of diagnostic, clinical assessment, or physical examination concerning non-traumatic knee pain. Articles on any age group were eligible, providing they described assessment/diagnosis of knee pain conditions seen in adolescents (e.g. studies on diagnosis of patellofemoral pain and ITBS in adults were eligible). Articles describing treatment only were excluded. Endnote version X9.1.1 was used to include or exclude articles.

Potentially eligible articles were independently screened by title/abstract and full text by one author (CG). Data were independently extracted and evaluated by discussion by two authors (CG and MES) into data extraction forms based on the Cochrane data extraction forms. We extracted data on study characteristics, prevalence of conditions, risk factors, clinical history and test for each diagnosis in the articles. Any discrepancies between forms were evaluated through discussion in the group.

#### Development process of the SMILE tool

Information from the review was then synthesised in a summary for each condition (see Additional file 2 and methods). This was supplemented by Brukner and Khan’s Clinical Sports Medicine 5TH edition, and input from international experts in sports medicine (published authors with more than five years of clinical experience), and GP’s with a special interest in sports medicine.

This information was then transformed into the SMILE tool. Table 1 shows an overview of the boxes included in the SMILE tool. Feedback on the content, layout, and text/frames used for the first version was sought from international experts and from medical doctors in a pilot test. The process from the first draft of the tool to the final version is shown in Additional file 2.

**Table 1:** Contents of SMILE tool

Overview of boxes in the SMILE tool	
Non-traumatic onset of knee pain	The first question is related to the onset of knee pain e.g. if the onset is non-traumatic with symptom getting worse and with no traumatic event at that point of time
Symptoms and pain localisation	Pain during loading activities Pain outside the knee joint Pain anterior on the knee ➔ where on front of the knee (tuberosity of the tibia, lower pole of the patella or around/behind the patella) Pain lateral on the knee or at the distal thigh Pain on the medial side of the knee Pain on the posterior side of the knee
Pictures of pain localisation	Each diagnosis is presented with a picture of the precise pain localisation
Tentative diagnosis – information boxes of each diagnosis	Pain localisation on palpation Epidemiology with sex differentials and age range
Differential diagnoses	Consist of the most important diagnoses that may not be missed in the clinic. Their symptoms and clinical characteristics

### Step two: validation of the SMILE tool

**Fig. 1:**
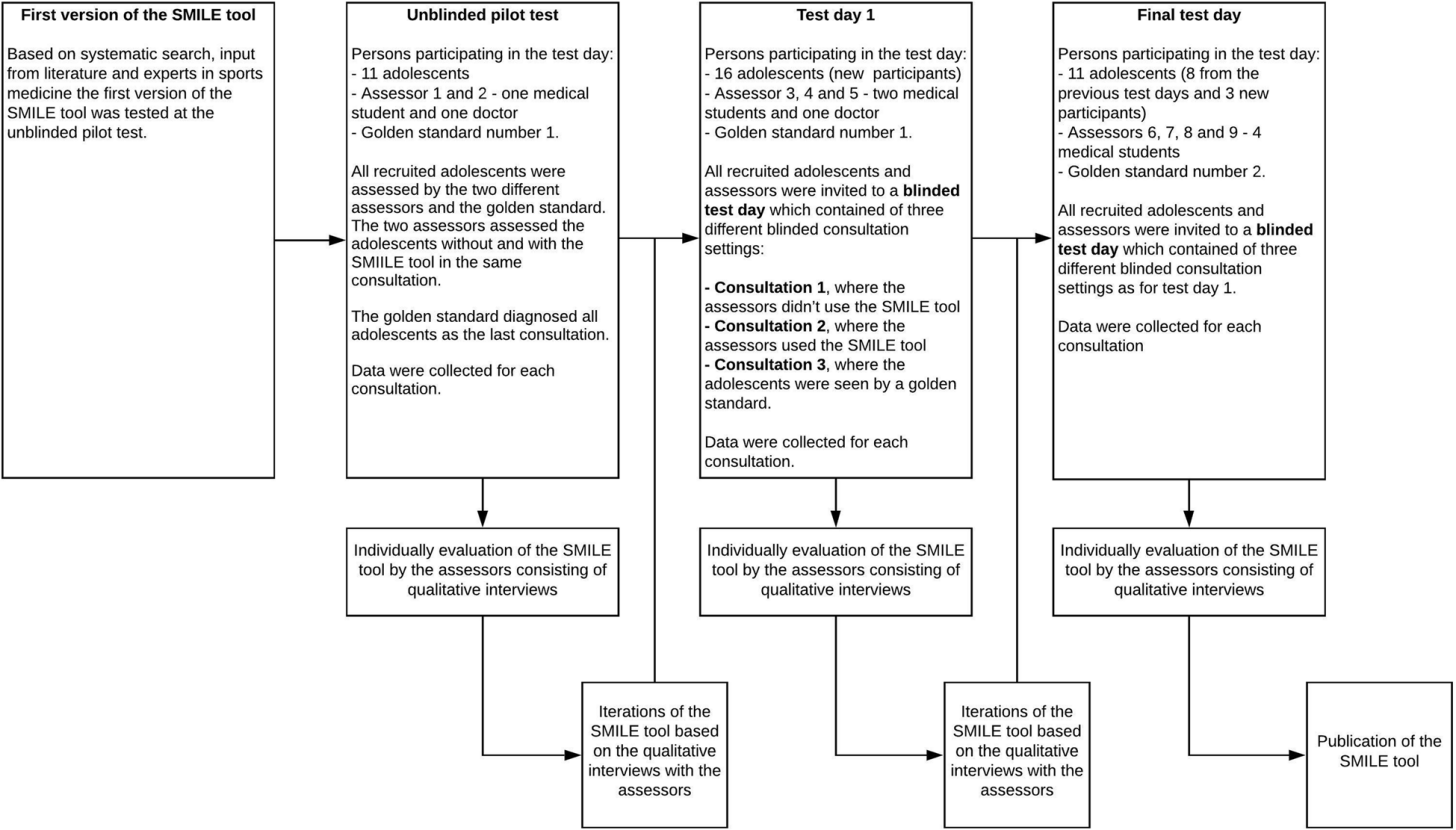
Overview of the three test days

After an initial pilot evaluation of the SMILE tool with one medical doctor and one medical student, we modified the layout based on feedback, and subsequently assessed and iterated the SMILE tool on two different test days. The content of the different test days and how we optimised and evaluated the SMILE tool after the test days is described in Fig. 1.

#### Recruitment of participants with knee pain

Inclusion criteria were adolescents (both sexes) aged 10–18 years, with a non-traumatic onset of knee pain. If adolescents had a traumatic event leading up to their onset of knee pain or had undergone surgery on the knee, they were excluded.

We recruited participants through flyers, videos on social media (Facebook and Instagram) and from local sports clubs.

#### Recruitment of medical doctors and medical students

The assessors were medical doctors or medical students recruited from the Center for General Practice and through our professional network. The medical students were required to be in their final year of medical educated needed to become a doctor in Denmark. We included medical students because the target end-users are inexperienced medical doctors without specific training/education in sports medicine or musculoskeletal disorders. We aimed that the SMILE tool would be feasible for medical doctors, independent of clinical experience with non-traumatic knee complaints in adolescents. Special care was taken to include assessors with varying interests in sports medicine. We included nine medical doctors and medical students (two younger medical doctors and seven medical students) as assessors in our study. Separate assessors were used at each session to ensure they were not familiar with the tool before assessments.

We recruited two specialists to serve as gold standards. The first (JLO) participated in the pilot test and test day 1, while the second (MSR) participated in the test day 2. Gold standard 1 is a specialist in rehumatology and sports medicine with 15 years of clinical and scientific experience in diagnosing and treating adolescents with knee pain [15]. Gold standard 2 is a physiotherapist and an experienced clinical researcher with a specific clinical and research interest in adolescent knee pain [16].

#### Examination of the SMILE tool through three different test days and data collection

Assessors received no training on the SMILE tool or its content, aside from a 1-min introduction. Assessors were interviewed regarding their perception of using the SMILE tool, and any potential areas of improvement. At each consultation assessors documented baseline characteristics (name, age, months with knee pain), diagnosis without and with the SMILE tool, and time spent on the consultation (see Fig. 1). Participants completed self-report questionnaires on knee pain duration, sports participation, and the Knee injury and Osteoarthritis Outcome Score (KOOS child) which has previously been used in adolescent populations with overuse related injuries [17]. Figure 1 gives an overview of the different test days and the data collection.

### Statistical analysis

Baseline characteristics for adolescents were calculated using descriptive statistics. To test the validity of the SMILE tool we calculated the percentage agreement without and with the SMILE tool between the diagnoses given by the assessors and the gold standards diagnoses. We tested the inter-rater reliability of the SMILE tool using Fleiss kappa statistics and McNemar’s test to determine differences in proportion correct diagnoses with and without the SMILE tool. Descriptive statistics were performed using Microsoft excel version 16.34. Fleiss kappa and McNemar’s test were calculated in R version 3.5.3.

## Results

### Development of the SMILE tool

**Fig. 2:**
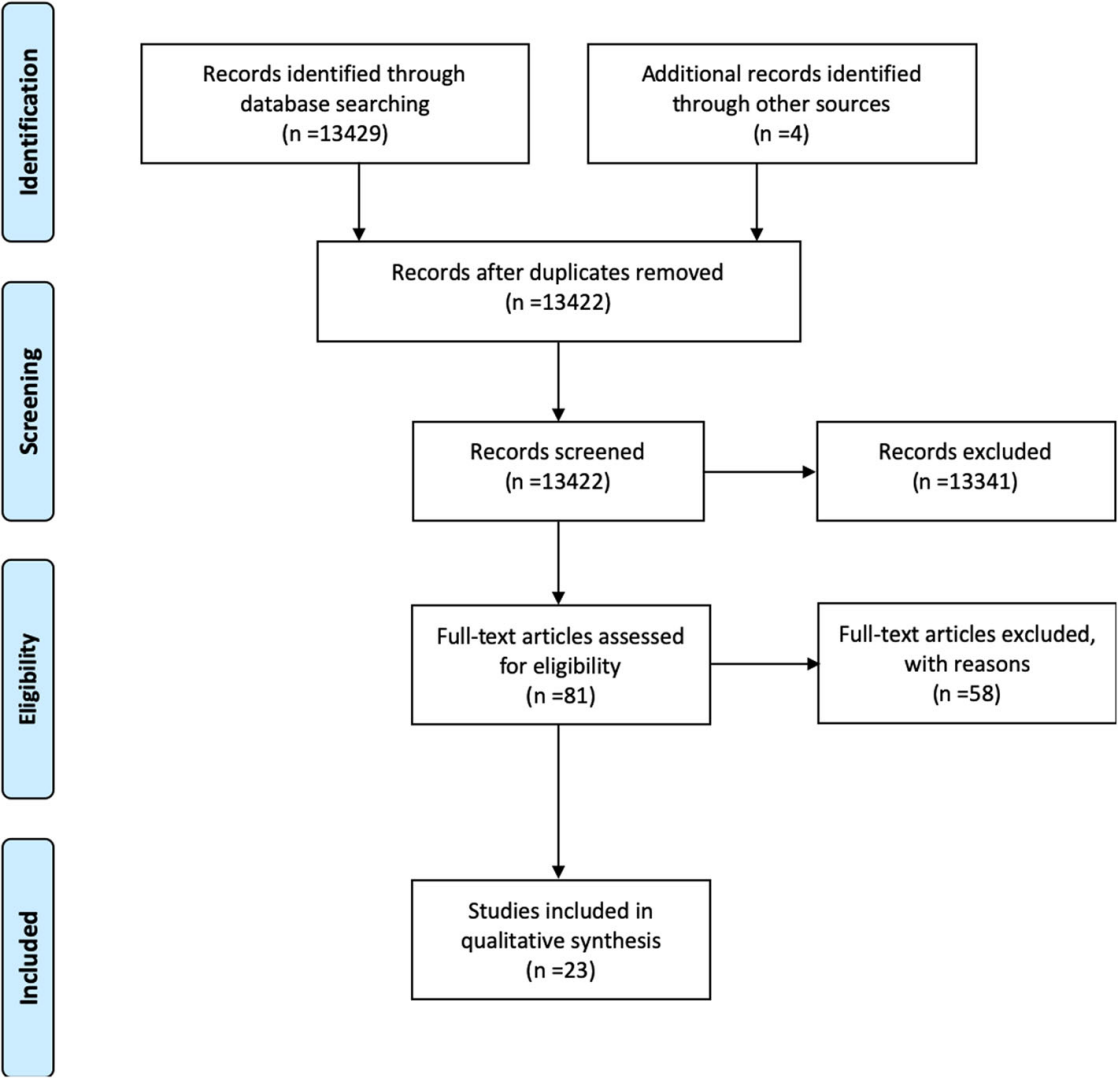
Prisma flowchart of the systematic literature search results from Medline

The systematic search revealed a total of 13,429 articles of which 81 were screened by full text. After full-text screening 23 articles were eligible for data extraction (Fig. 2).

**Fig. 3:**
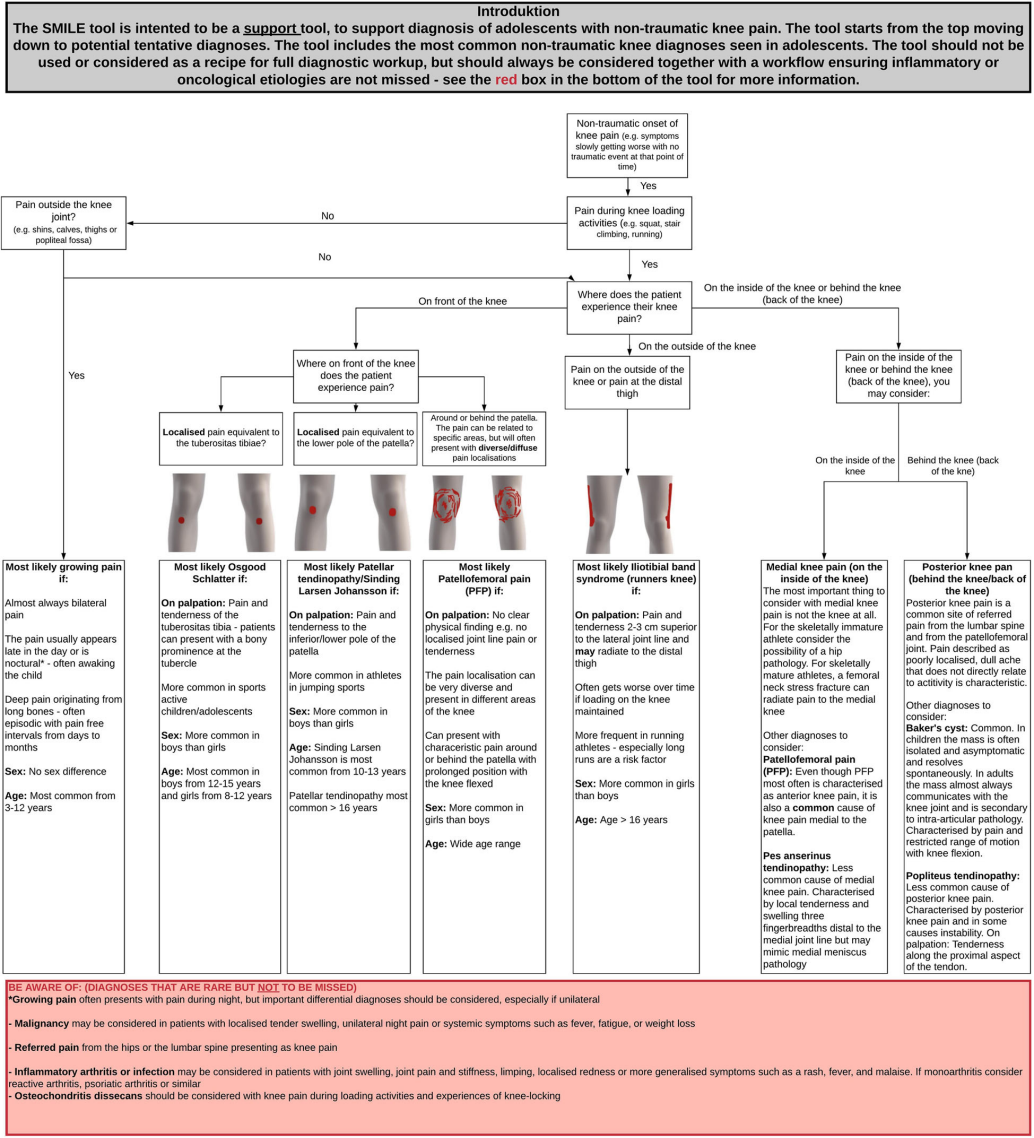
The final version of the SMILE tool

Based on the input from experts, the first version of the SMILE tool included the most common complaints which were growing pain, Osgood Schlatter, Sinding Larsen Johansson disease, Patellar tendinopathy, patellofemoral pain, and Iliotibial band syndrome as the main diagnoses in the tool (Fig. 3). We subsequently included the following differential diagnoses which were mentioned as important in the included articles and following feedback from experts and clinicians: Pes anserine tendinopathy, Baker’s cyst, popliteus tendinopathy, referred pain, inflammatory arthritis, osteochondritis dissecans, infection, and malignancy (Fig. 3). Figure 3 shows the final version of the SMILE tool. Detailed description of the development process is in Additional file 2 and methods section.

### Characteristics of participants and assessors

In total 27 adolescents (aged 10–17) participated (see Table 2). Of the 11 participants on test day 2, eight (72.7%) participated in one of the previous test days and were assessed by the two gold standards on separate occasions.

**Table 2:** Baseline characteristics of study participants for the first test day and second test day

Baseline characteristics	Test day 1	Test day 2
Number of participants (n)	16	11
Age (mean ± SD)	13.4 ± 1.9	13.6 ± 1.5
Sex (female, %)	81.3 (*n* = 13)	63.6 (*n* = 7)
Height (mean ± SD)	165.1 ± 11.7	168.1 ± 12.6
Weight (mean ± SD)	58.2 ± 21.7	60.8 ± 19.7
Months with knee pain (mean ± SD)	24.2 ± 12.8	22.8 ± 16.9
Weekly hours of sports participation (mean ± SD)	5.8 ± 2.6	6.3 ± 2.0
KOOS-Child Pain (0–100)	59 ± 14	68 ± 18
KOOS-Child Symptoms (0–100)	83 ± 14	85 ± 12
KOOS-Child ADL (0–100)	88 ± 8	92 ± 9
KOOS-Child Sport/Rec (0–100)	56 ± 19	65 ± 20
KOOS-Child QOL (0–100)	44 ± 19	56 ± 22
Participated in previous test days (%)	0 (*n* = 0)	72.7 (*n* = 8)

The seven assessors (three female and four male) had 0–4 years of clinical experience (mean 2.4 ± 1.5). Six were medical students in the final year of their medical education. One was a medical doctor with authorisation to practice. One had a special interest in musculoskeletal pain. The assessors came from 2 different Universities in Denmark.

### Agreement with the gold standards on test day one and test day two

Agreement with the gold standards increased with the use of the SMILE tool on both test days, with 77.3% percent agreement with the final version of the SMILE tool (Table 3).

**Table 3:** Agreement with gold standards for test day 1 and test day 2

	Average agreement between assessors and gold standards without the SMILE tool (%)	Average agreement between assessors and gold standards with the SMILE tool (%)	Change in percentage (%)	Relative increase in correct diagnoses	McNemar’s test (*P*-value)
Test day 1	18.8 (95% CI 12.8–37.3)	47.9 (95% CI 40.0–68.3)	29.2	2.5	< 0.001
Test day 2	22.7 (95% CI 10.3–35.1)	77.3 (95% CI 64.9–89.7)	54.6	3.4	< 0.001

One participant had differing diagnoses by the two assessors used as gold standard on the different test days; PFP and OSD respectively. Among the junior assessors, seven out of seven diagnosed the participant with OSD without the tool on the two test days. The participant reported pain directly at the tibial tuberosity with a bony prominence. Therefore, the participant was considered to have OSD.

### Inter-rater reliability and agreement with the gold standard for test day one and test day two

Inter-rater reliability increased with the use of the SMILE tool on both test days, with a moderate agreement on the final version of the SMILE tool. On the final test day (test day 2) Fleiss’ kappa increased from *poor* agreement without the tool k = − 0–04 (95% CI, − 0.12 to 0.04), to *moderate* agreement with the SMILE tool k = 0.56 (95% CI, 0.40 to 0.72).

## Discussion

### Summary

To our knowledge, this is the first study to develop and initially evaluate a support tool for clinical practice to improve management of adolescents with non-traumatic knee pain. The percentage of correct diagnoses and reliability reached nearly 80% using the final version of the tool. This reflected an approximate fourfold increase in junior medical doctors’ correct diagnoses, compared to without the SMILE tool. The reliability of the final version of the SMILE tool between assessors was moderate.

### Strengths and limitations

Our validation is based on a relatively small sample size and external validation is needed. We purposefully recruited medical students to reflect inexperienced doctors. This choice may mean that the different in correct diagnosis may be larger among this sample compared to a sample of more experienced medical doctors. We cannot generalise our results to other professions in sports medicine, orthopaedics or other areas where this patient population consults. Further each participant’s cause of knee pain was unknown at recruitment and we were unable to recruit according to specific diagnoses and therefore certain conditions, i.e. growing pain, were not represented among the participants. However, our primary focus was on non-traumatic knee injuries, which were well represented and for which we showed high validity.

Our testing was not conducted as part of a normal clinical day of clinical practice. This tool was designed for non-specialist doctors (GPs). The assessors had 10 min for each to ensure comparability to clinical practice. The flow of patients was not identical to a normal clinical day as all patients had knee pain, which made it easier for the assessors to focus only on knee pain. Future studies may focus on the implementation of the SMILE tool in a clinical practice setting including the need for more explicit guidelines on when to refer patients for example if oncologic or inflammatory etiologies are considered. Finally, this tool was designed for GPs, and has not yet been tested with other types of medical doctors. The tool was largely tested by medical trainees in a general practice setting to specifically target junior doctors in this setting. In this study we recruited patients with non-traumatic knee pain. The patients included, had many of the diagnoses outlined in tools, as we developed this to consider the most common diagnoses. Further research is needed to evaluate whether the tool can adequately support clinicians with patients with more diverse conditions, and correctly rule out serious pathology as this has not been evaluated in this initial study.

### Comparison with existing literature

Diagnosing knee pain can be challenging, especially during adolescence where growth related injuries (Osgood Schlatter, Sinding-Larsen Johansson) are common and unique to this population. It is also important to differentiate growing pains and growth related non-traumatic knee pain. In this study, the assessors only diagnosed about 20% of adolescents correctly compared to specialists (gold standards) without using the SMILE tool, with a poor reliability between assessors. Given the importance of diagnoses for treatment [12, 14], this tendency of low diagnostic accuracy is concerning.

Diagnostic clarification is important for adolescents when consulting for knee pain [14]. However, GPs find it challenging to give the correct diagnosis [2, 13]. We know that non-traumatic knee complaints are overlooked compared to traumatic knee complaints [3, 4]. Our assessors focused on traumatic causes of knee pain when they assessed patients without our tool (data included in Additional file 3). This underlines younger GPs’ or junior medical doctors’ need for guidance when assessing adolescents with non-traumatic knee pain.

Current available clinical tests primarily focus on a single diagnosis [5, 18–23]. Our simple SMILE tool does not require any clinical tests except palpation and pain localisation, which was highlighted in the interviews by the assessors that this made the SMILE tool extremely user-friendly without any training. Furthermore, the assessors highlighted that this tool is easy to use and time efficient, which may facilitate implementation into a GP consultation. Most importantly, it does not need any pre-training or specialist introduction which may facilitate use and implementation.

We aimed to create a tool that includes the most common presentations of non-traumatic adolescent knee pain, as ease-of-use and time requirements are important factors for GPs and medical professionals [24, 25]. The high level of agreement between our SMILE tool and the gold standard suggest that despite having several diagnoses it has a high validity. We showed that our inexperienced assessors improved their diagnostic accuracy almost 4 times with the assistance of the SMILE tool. Further, their focus shifted from thinking about traumatic causes of knee pain, even in presentations without trauma, to thinking about non-traumatic causes (data shown in Additional file 3). This shift may be favourable in general practice, as non-traumatic causes of knee pain are the most prevalent in adolescents [3].

### Implications for research and/or practice

This tool was developed for a general practice setting and therefore we developed a simple tool feasible to use in the setting of a 10-min consultation without previous knowledge of the patient. This tool is primarily made for GPs who have a wide range of different patients daily, where it is still necessary to maintain a high level of expertise in all topics to meet the patients’ expectations and provide the appropriate management. Our SMILE tool gives an easy overview of the most common non-traumatic knee complaints seen in adolescents without the need of much introduction and can thereby easily fit into a general clinical practice setting with a variety of different patients. Despite being designed for general practice setting, this tool could influence other medical specialities including orthopaedic surgery and pediatric rheumatology. Patients are often co-managed between specialities and if correct diagnosis is achieved in initial assessment in general practice, this could lead to more relevant referrals and targeted treatment.

## Conclusion

We developed and performed initially validation of the SMILE tool for non-traumatic knee pain in adolescents. This development study was the first step in the process of developing a tool for diagnosing knee pain. The results indicate it’s potential, however, the external validity should be evaluated in clinical practice with clinicians. Our simple tool is quick to use and covers the different causes of non-traumatic knee pain in adolescents and can be used to support clinical practice.

## Supplementary Information

**Additional file 1.**

**Additional file 2.**

**Additional file 3.**

## Data Availability

Data can be made available upon request to the authors.

## Abbreviations

GP: General practitioners
SMILE tool: Sorting non-trauMatIc adoLescent knEe tool
KOOS: Knee injury and Osteoarthritis Outcome Score
