# Supplementary material for: Development and validation of the Sorting non-trauMatIc adoLescent knEe pain (SMILE) tool – a development and initial validation study
Source: Pediatr Rheumatol Online J. 2021 Jul 6;19:110. doi: 10.1186/s12969-021-00591-5 (PMC8259444; doi:10.1186/s12969-021-00591-5)
Supplement: Supplementary file 1 — Additional file 1. [file 12969_2021_591_MOESM1_ESM.docx]

# Additional file 1.

| Knee pain/injuries | Children/adolescents | Assesments/diagnosis |
| --- | --- | --- |
| “Knee pain”  Patellar tendin  Patellar tendinopathy*  Apophysitis  Jumpers knee  Osgood  Schlatter  Patellofemoral Pain Syndrome  (Patella/Patellofemoral Joint)  Patella Ligament  (Patellofemoral Arthralgia)  Chondromalacia patella  Tibial tubercle avulsion  Sinding Larsen Johanssons  Hoffa | Child  Kids  Adolescent  Teenager / teen  Young  Athlete  Juvenile  Junior  Paediatric  Immature | Presentation  Sensitiv*  Sensitivity or specificity  “Likelihood ratio”  “Post-test Probability”  “Predictive value”  History  Diagnos*  Physical exam*  Clinical assessment  Clinical exam |
