# Supplementary material for: Development and validation of the Sorting non-trauMatIc adoLescent knEe pain (SMILE) tool – a development and initial validation study
Source: Pediatr Rheumatol Online J. 2021 Jul 6;19:110. doi: 10.1186/s12969-021-00591-5 (PMC8259444; doi:10.1186/s12969-021-00591-5)
Supplement: Supplementary file 2 — Additional file 2. [file 12969_2021_591_MOESM2_ESM.docx]

# Additional file 2: Supplementary materials and methods– process from first draft to a final tool

This process document is made to document all steps from the absolutely beginning of the nontraumatic diagnostic tool to the final version of the nontraumatic diagnostic tool.

## Step 1.

### First draft of tool – version 1.0

First step of developing the diagnostic tool, was to make a short summary of every condition based on the data extraction from the systematic literature search. A table was made separately for each condition with relevant references where the information for each condition came from.

| Growing pain^23^ | The most common cause of childhood musculoskeletal pain – prevalence range between 3-37% of children with an unknown etiology  Characteristic with non-articular pain, 2/3 of children is located in the shins, calves, thighs or popliteal fossa – almost always bilateral  The pain usually appears late in the day or is nocturnal – often awaken the child with a pain duration between minutes to hours – by morning the child is almost always pain free  No objective signs on inflammation on physical examination |
| --- | --- |
| Osgood Schlatter^3, 9-11, 17, 21^ | Common condition, boys between 12-15 and girls between 8-12  Traction apophysitis and traumatic avulsion of the secondary ossification center of the tibial tuberosity  Gradual onset of pain, swelling and tenderness in the tuberositas tibia region on palpation  Pain is mild and intermittent initially  Pain exacerbates after sporting activity involving jumping, running and/or direct contact (kneeling)  **Objective test**  Tenderness, local swelling and prominence in the area of the tibial tuberosity +  Pain can be reproduced with extension of the knee against resistance |
| Sinding Larsen Johansson^10, 14, 17, 21^ | Traction apophysitis of the inferior patellar pole  Children present at the age of 10-12  Knee pain localised to the inferior patella  Knee pain presents with knee loading activities such as running, jumping, climbing stairs |
| Patellar tendinopathy^1, 10, 20, 21^ | Repetitive jumping sports such as basketball and volleyball  Male predominance  Anterior knee pain that increases with activity  Well localized pain and tenderness of the inferior pole of the patella  Pain with prolonged sitting, squatting and stair climbing  Tendon pain occurs while loading and usually stops almost immediately when the load is removed |
| ITBS^1, 4, 10, 17, 22^ | Running and jogging  Each time the knee flexes and extends during an activity such as running the iliotibial band rubs across the lateral epicondyle 🡪 inflammation of the lower portion of the band  Genu varum has been expected as an intrinsic factor  Diffuse lateral knee pain that worsens with long or hilly runs or with going up and down stairs  Athletes with ITB syndrome typically complain of a sharp or burning pain roughly 2 cm superior to the lateral joint line. The pain may radiate proximally or distally, and in less severe cases, the pain may quickly subside upon cessation of activities. Often pain will occur as activities proceed. It is not uncommon that the athlete will experience popping on the lateral aspect of the knee with activities  ITB syndrome is a clinical diagnosis and most often additional diagnostic studies are not necessary. It should be suspected in overuse and nontraumatic cases of knee pain where rest has not been helpful  Most common in girls versus boys |
| Patellofemoral pain^1, 2, 5-8, 12, 13, 15, 18, 19^ ^16^ | PFPS is a **clinical diagnosis** and treatment can be initiated without imaging  Chronic anterior or retro patellar knee pain that intensifies with activity or prolonged sitting  More common in girls than boys  Common among adolescents and young adults  The typical history is an athletic young woman who denies acute trauma but reports knee pain in weight-baring activities – month or years of bilateral pain that worsen if climbing stairs or hills + anterior knee pain with prolonged sitting  Tenderness on palpation of the patella facets and a small effusion. Assessment of functional movement should be evaluated by specific test – squatting, running, jumping  If asked to point out the location patients may place their hands over the anterior aspect of the knee or draw a circle with their fingers around the patella (“the circle sign”)  Apprehension test and Clarke’s sign  Mild effusion and tenderness around the medial and lateral retinacular tissues, Tight hamstrings, Patella grind positive |

**Table S2.** Shows the information of each condition based on literature search (PubMed).

Based on the data extraction from the systematic literature search in Medline, we made the first draft of the tool to look like this: **Figure S1.** First draft of nontraumatic diagnostic tool version 1.0. Only based on original research and narrative articles from the systematic search on PubMed.

### Evaluation in the team – version 1.1

The first draft of the diagnostic tool was evaluated together in the team consistent of: Sinead Holden (supervisor), Marina Elmelund, Michael Skovdal Rathleff (supervisor) and Clara Guldhammer (first author). Based on discussion we made the following changes to the tool:

- No clear cut between children and adolescents in the top of the tool (we deleted the box with “children/adolescents” and “adolescents/adults)
- Sitting was removed from the box with loading activities as they are more specific symptoms rather than general
- After the box with “Anterior knee pain” we added a new bow if the answer to that was “yes” where we ask if the knee pain is localised or diffuse, so it is easier for the doctor to separate between PFP and the other diagnoses
- Tendonitis was changed to tendinopathy throughout
- Instead of having Sinding Larsen Johansson (SLJ) and Patellar Tendinopathy (PT) in different boxes we decided to have them in the same box as they are similar to each other and have to be manage in the same way regarding treatment
- We added “pain with prolonged sitting” to the SLJ/PT and Patellofemoral pain (PFP) box
- We have added a “be aware box” with information on diagnoses that aren’t rare, but not to be missed in the clinic

**
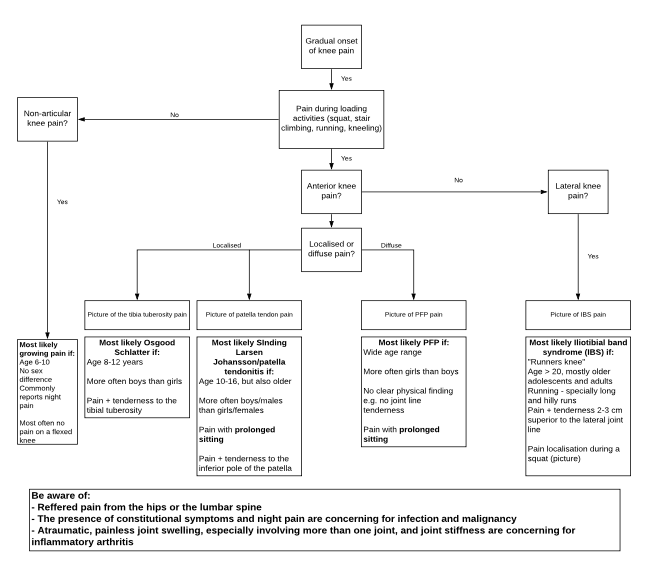
**

**Figure S2.** Nontraumatic diagnostic tool version 1.1 (after the first evaluation in the team)

### Evaluation in the team part 2 – version 1.2

The second evaluation on the tool was made in the team as above.

- We added a picture of the pain localisation to every diagnosis
- To the box with OSD we added “More common in sports active children and adolescents” as this is well-known in the literature and an important characteristic to OSD


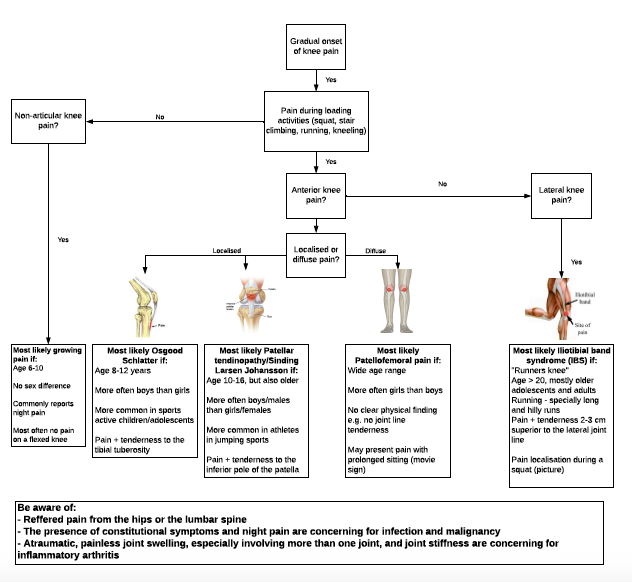


**Figure S3.** Nontraumatic diagnostic tool version 1.2 – this tool was sent to international experts.

To get as much feedback as possible from different perspectives the team decided to ask international experts to evaluate the tool. Nontraumatic tool version 1.2 was then sent to two different international experts in sports medicine and musculoskeletal pain.

## Step 2.

### Inputs and feedback from international experts

The Nontraumatic tool version 1.2 was sent to two different experts with no contact to each other. The correspondence between the international experts and our team was through Email and Skype meeting with Michael Skovdal Rathleff as the contact person from our team.

### Iterations after expert inputs

Based on the comments from expert 1 and 2 we have made the following changes;

- Gradual onset 🡪 change to "non-traumatic on set" e.g. symptoms slowly getting worse with no traumatic event at that point of time
- We removed “kneeling” from the box with “pain loading activities” as it is more specific for OSD than in general
- To the box “Lateral knee pain” we added “or pain at the distal thigh” as pain at the distal thigh is an important pain localisation to recognise IBS
- We decided to keep “non-articular knee pain” as wording in the box leading down to “growing pain” as we think non-articular is a well-known term
- Epidemiology (age and gender) information in each diagnosis box was moved down in every box so pain localisation on palpation is the first information in each box
- To IBS we added “getting worse over time” as this is an important characteristic
- To the box about PFP we added “retro patellar or peri-patellar pain” and removed “the movie sign”

**
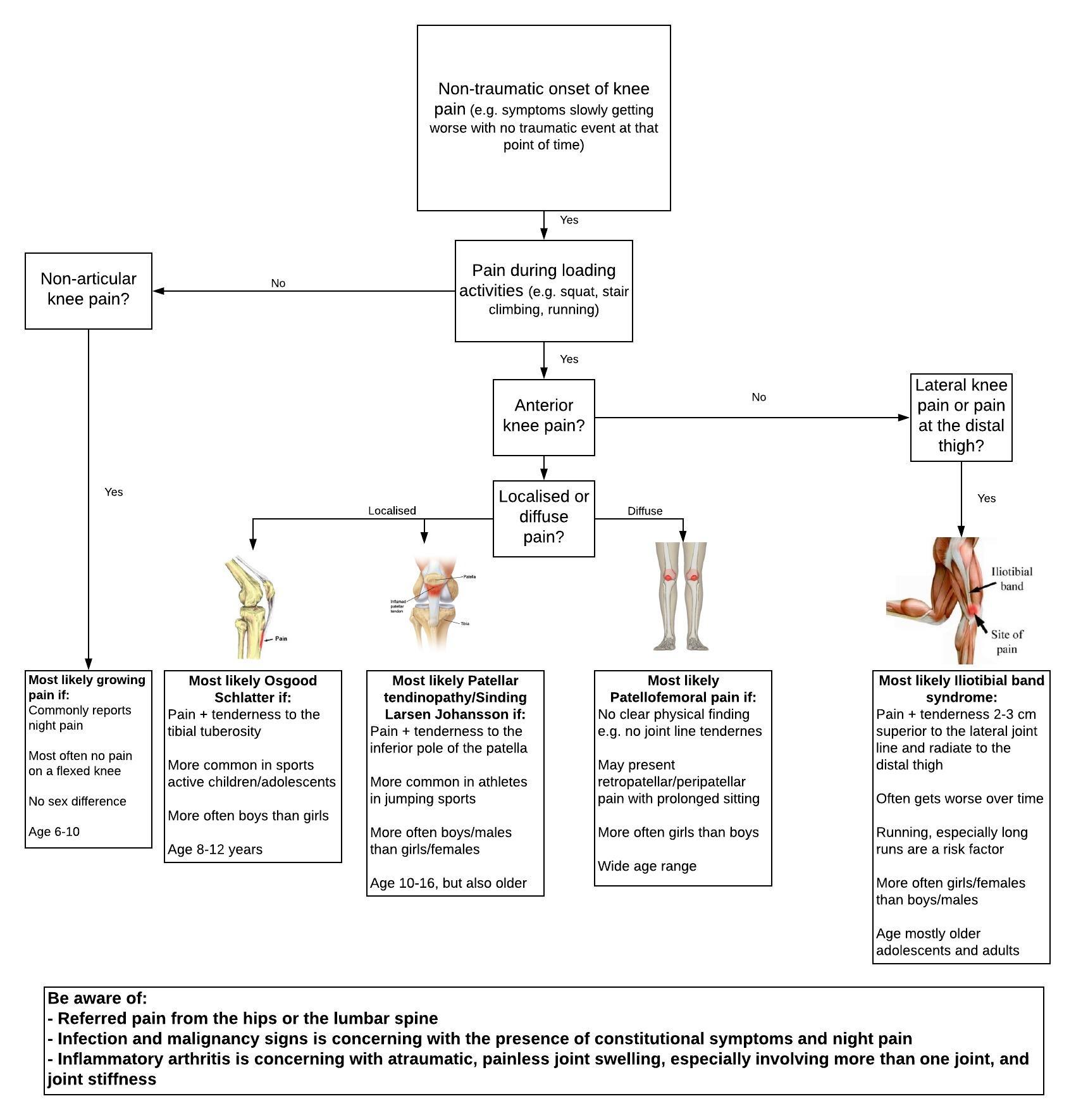
**

**Figure S4.** Nontraumatic diagnostic tool version 2.0 based on comments and feedback from expert 1 and 2.

### Evaluation in the team part 3 – version 2.1

- We made a path from non-articular knee pain if the answer is “no” back to “anterior knee pain” as some PFP patients only have pain with prolonged sitting and then can say “no” to pain during loading activities and then end up with nothing if the answer to “non-articular knee pain” is no as well
- We specified the box “anterior knee pain” and added “pain localised on front of the knee” to make sure that the doctors aren’t in doubt about the definition of “anterior knee pain”
- To differentiate we changed “localised” to “pain on palpation of TT”
- We added more information to the “be aware box” and painted the text with red as a “aware” sign
- In the first dot of the “be aware box” we added “presenting as knee pain” in the end of the sentence
- We removed “most often no pain on a flexed knee” and added “deep pain originating from long bones” to the box regarding growing pain
- We added “most common” to boxes with age range as per sex differences
- “***may*** radiate to distal thigh” was added to the IBS box

**Figure S5.** Nontraumatic diagnostic tool version 2.1 based on comments and feedback from Sinead and Michael

### Evaluation in the team part 4 – version 2.2

- In the box “Pain during loading activities” add “knee” before loading activities
- From reading again I think the figures with pain localisation ruin the flow right now. Wonder if we should put in label of diagnosis already in this text box? “i.e. most common pain localisation in patellar tendinopathy” otherwise the “most common” part of the sentence is without meaning
- Spelling error in “localisation”
- Diagnosis that “are” and not “is”
- Inflammatory arthritis be aware of atraumatic, painless….
- In the box “Pain during loading activities” we added “knee” before loading activities
- Pictures of pain localisation were incorporated in the end-boxes for each diagnoses so they didn’t stop the flow in the tool
- “Inflammatory arthritis, be aware of atraumatic, painless” was added to the “be aware of box”
- Misspellings and grammatic errors were corrected throughout

##

**Figure S6.** Nontraumatic diagnostic tool version 2.2 based on comments and feedback in the team

## Step 3.

### Inputs and feedback from expert in sports medicine (rheumatologist)

Based on comments from the rheumatologist we made version 2.3 of the tool.

- **Box with “non-articular knee pain”: Is this based on passive movement or? Need some guidance to help the GP what you mean by “non-articular” – how to differentiate between articular and non-articular? e.g. pain at the TT is not articular –** we changed to “pain outside the knee joint e.g. shins, calves, thighs or popliteal fossa)
- **Box with “anterior knee pain”: “Is the knee pain in front of the knee?” – AKP could be consider a diagnosis –** we changed to “pain anterior on the knee” as “anterior knee pain” is a specific diagnosis known by Danish medical doctors, so to clarify that this box doesn’t consider the specific diagnoses we made the change
- **Box with “lateral knee pain or pain at the distal thigh”: lateral knee pain (its because it would lead to a dead end if the answer is No to anterior knee pain and No to lateral (i.e. if they have medial/lateral). Then you could include a box to Consider differential diagnosis / guidance for medial / posterior pain. Can be  a small box ‘if medial pain think X, if posterior think Y – We made a box with ”medial and/or posterior knee pain consider” and then a list with the different diagnoses to conside -** We incorporated this step so there was not a dead end in the tool
- **Add age filter e.g. > 16 then prob PT, but otherwise branch to OSD/SLJ on pain localisation**
- **Box with “Growing pain” (GP) : Be careful, night pain in the knee will be a red flag for** we incorporated a little red star to “nocturnal pain” with an explanation of GP presenting as nocturnal pain and how to differiate between GP and more serious diagnoses

**Figure S7.** Nontraumatic diagnostic tool version 2.3 based on feedback from Jens and discussion in the team

## Step 4.

### Inputs and feedback from Danish general practise doctors with an interest in sports medicine – version 2.4

The English version was translated into a Danish version. The Danish version of the nontraumatic diagnostic tool was sent to the 4 doctors in general medicine.

**Figure S8.** Nontraumatic diagnostic tool version 2.3 in Danish

### Changes made from version 2.3 – 2.4 (feedback and comments from Danish doctors)

- **Include osteochondritis dissecans in “be aware” box** – this is added to version 2.4
- **Localised or diffuse pain – include the word “now” so that the doctors know it is the present knee pain** – we changed to “is the knee pain localised or diffuse now?”
- **Include reactive arthritis in the part with inflammatory knee pain** – this is added to version 2.4
- **Write “unilateral” in the sentence with GP in the “be aware box”** – we changed the sentence from “should be considered, especially if not bilateral” to “should be considered, especially if unilateral”
- **Age with SL – should be 10-13 years** – changed throughout

**Figure S9.** Nontraumatic diagnostic tool version 2.4 in English.

The same changes were made in the Danish version:

**Figure S10.** Nontraumatic diagnostic tool version 2.4 in Danish.

### SLJ and PT – together in the same box or seperated?

Should SLJ and PT be together in the same box or separated was an issue we discussed in the team. We decided to ask the end users of the tool – the general practise doctors that they prefer, and they all prefer to have it together with the devise “keep it as simple as possible”. We therefore decided to have SLJ and PT together in our diagnostic tool. This might be changed in future editions, but for the test day it will end up in the same box and will be a focus point for the users on the test day.

## Step 5 – Unblinded pilot test

Version 2.4 in Danish was used at the test day.

**Figure S11.** Nontraumatic diagnostic tool version 2.4 in Danish tested at the test day

### Higlights from interview with doctor 1 and 2

- Both doctors agreed on that the tool was very easy to use and remember. There was a good flow from the top of the tool to the end – and no stop point where it doesn’t flow with a normal consultation
- The tool made it easier for the doctors to separate the different knee diagnosis. They both felt more comfortable to give a tentative diagnosis with using the tool
- Both doctors definitely think they will use the tool if it was available in a general practise setting
- Both of the doctors find it difficult with the box about “medial/posterior pain” – they both wished for a little more explanation to these conditions and how they differ from each other and the other diagnoses
- The pictures should be bigger, so they are easier to look at and use as a guide for the pain localisation

### Changes made to the tool after evaluation

- As both doctors think that the box with medial/posterior knee pain needed more explanation, we added information on the basic characteristics and how they differ from the other diagnoses
- Pictures with pain localisation in a better quality was a step that we before the test day knew should be better but didn’t have time to make. The pictures are now drawn in Navigate Pain and moved up from the information boxes, so they stand beside each other, so it is easier to differentiate between the diagnoses in relation to pain localisation
- We have made the “be aware box” red, so it is clearer that these conditions are serious and not to be missed in the clinic
- “on palpation” “Gender” and “age” are highlighted with bold type, so it is easy to find in the information boxes
- In the “be aware box” we have written the diagnoses with bold type

**English version 2.5**


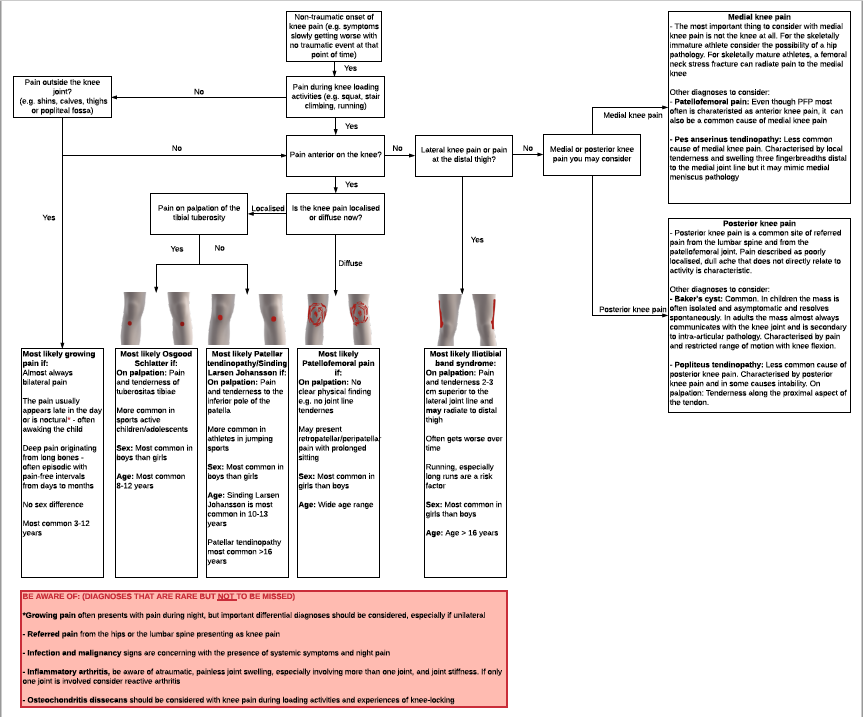


**Figure S12.** Nontraumatic diagnostic tool version 2.5 in English

**Danish version 2.5**

**
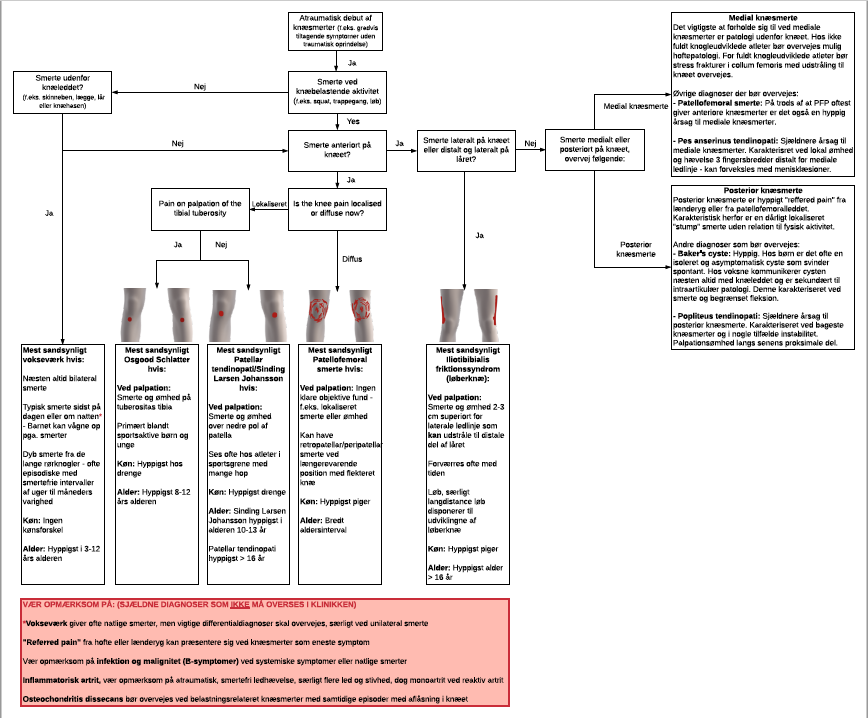
**

**Figure S13.** Nontraumatic diagnostic tool version 2.5 in Danish (tested at the test day 2 see below)

## Step 6 – Test day 1

Version 2.5 in Danish was used at the test day.

**
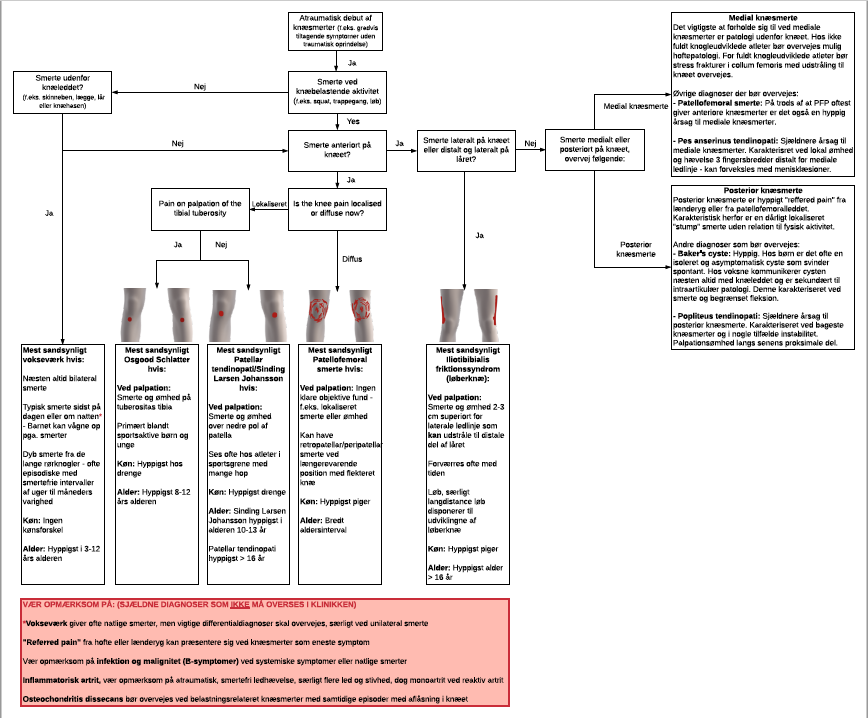
**

**Figure S14.** Nontraumatic diagnostic tool version 2.5 in Danish

### Higlights from evaluation and interview with doctor 1, 2 and 3

- All doctors agreed on that the tool was very easy to use and remember. There was a good flow from the top of the tool to the end
- The tool made it easier for the doctors to separate the different knee diagnosis. They all felt more comfortable to give a tentative diagnosis with using the tool
- Both doctors definitely think they will use the tool if it was available in a general practise setting
- 2 out of 3 doctors find the use of medical terms related to the pain localisation (anterior, lateral, medial, posterior) difficult in the end of the day when they have seen a lot of patients and asked if it could be in public language instead of medical language – as simple as possible
- All the doctors had some problems with the box “localised or diffuse pain” where they find it difficult to clarify together with the patients if their knee pain were localised or diffuse. Furthermore, based on the results, we can see that there were made some wrong diagnoses where the doctors inappropriately favoured localised pain over diffused pain.
- 2 of the doctors came with the idea that instead of having the pain localisation boxes beside each other with a yes/no solution, the tool could have an overall question about the pain localisation “Where does the patient experience their knee pain” and divide into anterior, lateral, medial and posterior in one line beside each other - 2 of the doctors experienced a "stop" in the flow if the patients answered no to anterior knee pain - I have attached Sabinas response below as Theis' was similar, just not as detailed as Sabinas.

### Changes made to the tool after evaluation with the doctors

Taking these comments into consideration we have tried to make 2 version

- **Version A**
  - Anterior, lateral, medial and posterior was changed to public language
  - Instead of asking "Is the knee pain localized or diffuse?” we changed to ask ”where does the patient experience their knee pain?” and with a guide where we want the patient to point out where they have their pain
  - We made three new boxes
    - 2 with localised pain (OSD and SLJ/PT) where we with bolt have tried to point out that it should be LOCALISED pain
      - **Localised** pain on the tuberositas tibiae?
      - **Localised** pain at the lower pole of the patella?
    - 1 with PFP where we try to explain that the pain is around or behind the patella, but also explain that that it can be related to single localisations or more diffuse with more pain localisations ”
      - Around or behind the patella. Can be individuals areas, but often more diverse pain localisation


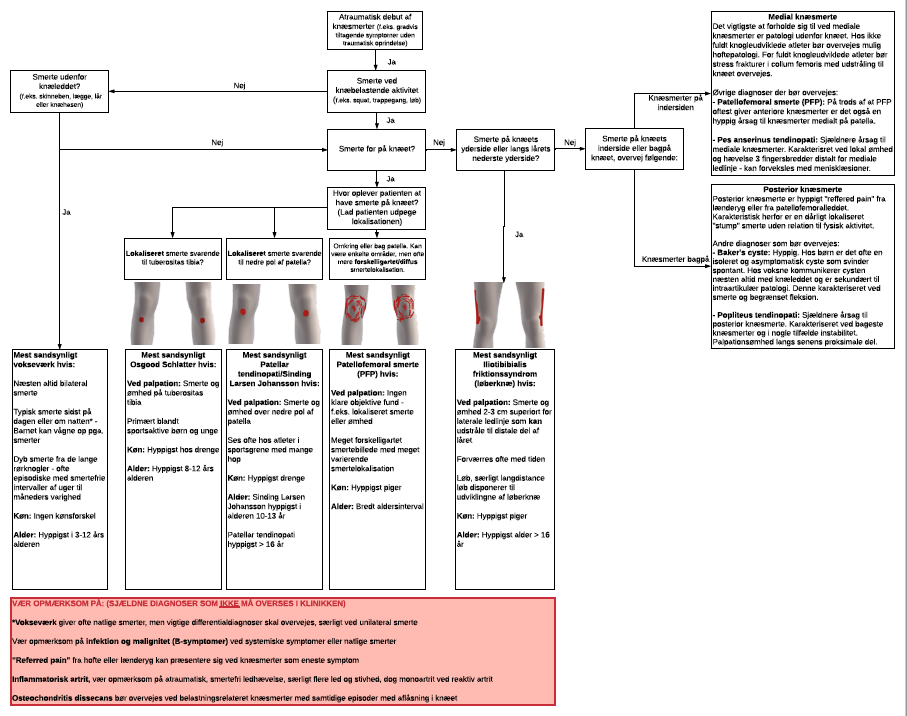


**Figure S15.** Nontraumatic diagnostic tool version 2.6 A in Danish

- **Version B**
  - Anterior, lateral, medial and posterior were changed to public language
  - After the box with ”Pain with knee loading activities” we made a new box where we ask ”Where does the patient experience their knee pain?” 🡪 after this box we devided into three possible answers (in front of the patella, outside of the patella, behind or on the inside of the patella)
    - **In front of the patella 🡪** Seperate in 1 arrow asking ”Pain in front of the knee”, which then devide into 3 arrows/boxes with localised pain equivalent to the tuberositas tibiae or the lower pole of the patella, or if it is more diverse related to PFP
    - **Outside of the patella 🡪** When the patient point of that it is on the outside of the patella the next box ask if the pain is related to the distal thigh or on the outside of the patella and then lead down to ITBS
    - **Behind the patella or on the inside of the patella 🡪** This is seperated into 2 arrows with ”inside of the patella” and ”behind the patella” which then devide into a box with medial knee pain and a box with posterior knee pain


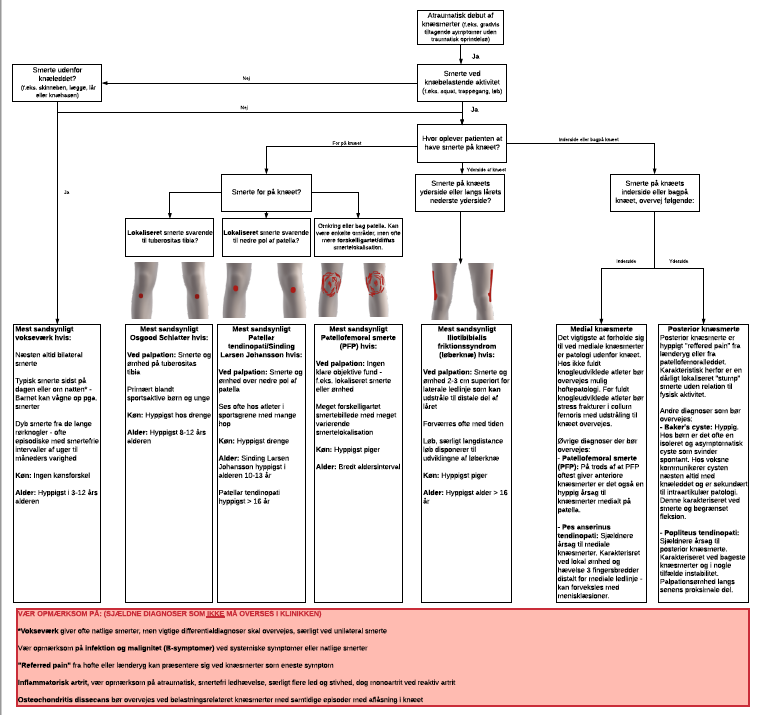


**Figure S16.** Nontraumatic diagnostic tool version 2.6 B in Danish

### Version 2.6 A or 2.6 B?

In the team we discussed whether we should go with version 2.6 A or 2.6 B in the next and final test day (final version of the tool). Based on the evaluation from the doctors and how we think the design looks best and is easiest to understand for the younger doctors, we decided to use version 2.6 B.

All in the team liked that all the diagnoses are “in line” in this version and the main question is “where does the patient experience their knee pain” – which then will divide into 3 ways to go. In the previous versions of the tool and in version 2.6 A it was a little biased that we knew a lot of the patients will have anterior knee pain and therefore was this step more prioritized than the other pain localisations. This is changed in the 2.6 B version where anterior, lateral, medial and posterior knee pain are in line with each other.

We made a small change to the 2.6 B version after the meeting, as we made the “anterior knee pain” box to a question: “Where on front of the knee do you have pain?” which lead down to questions to differentiate between OSD, SLJ/PT and PFP.

After these changes we send the version 2.6 B to the doctors who participated in the test day 2 and asked what they think of the changes. All agreed that they like the changes and were similar to what they had thought of in the evaluation.


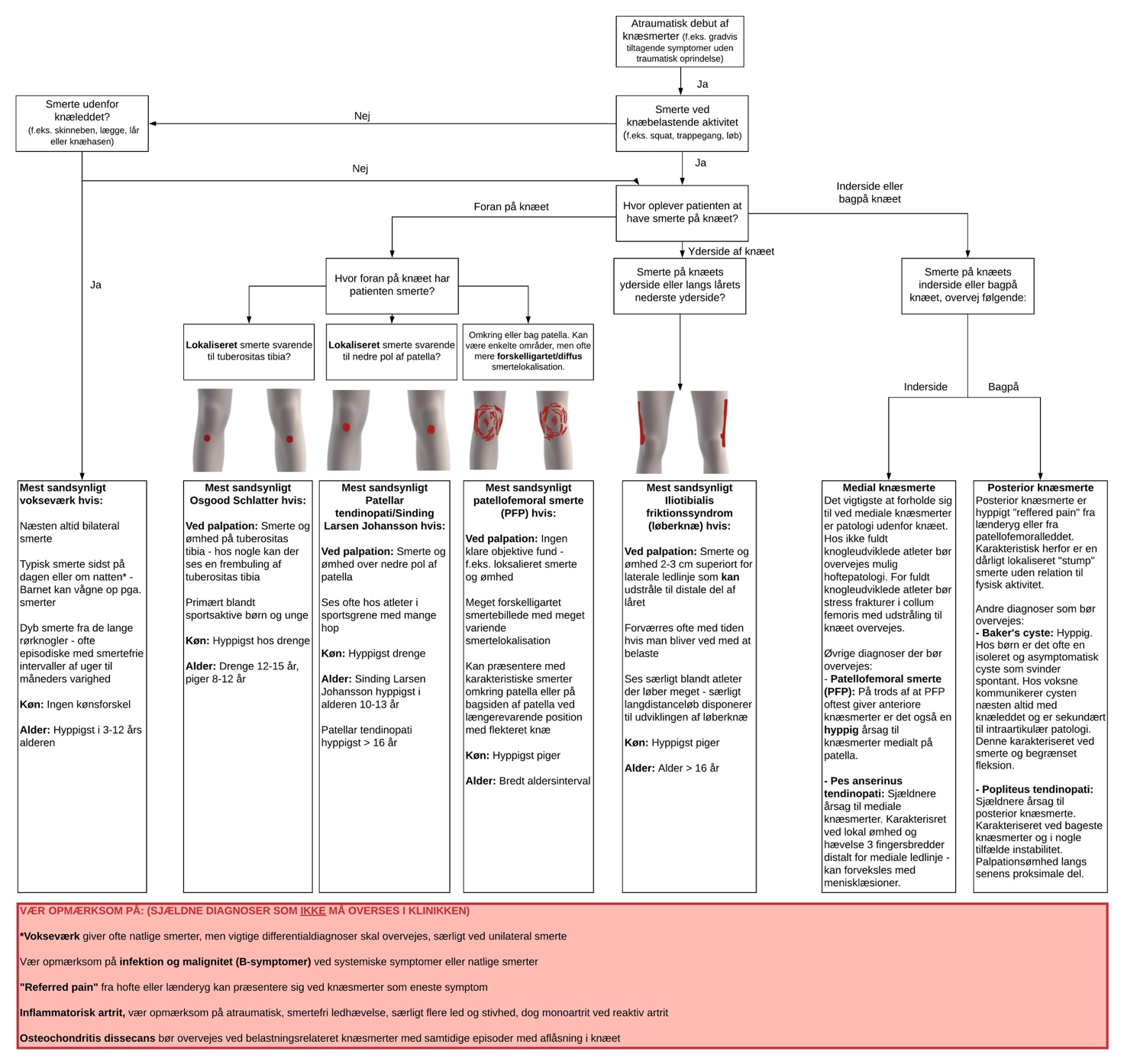


**Figure S17.** Final version in Danish

**
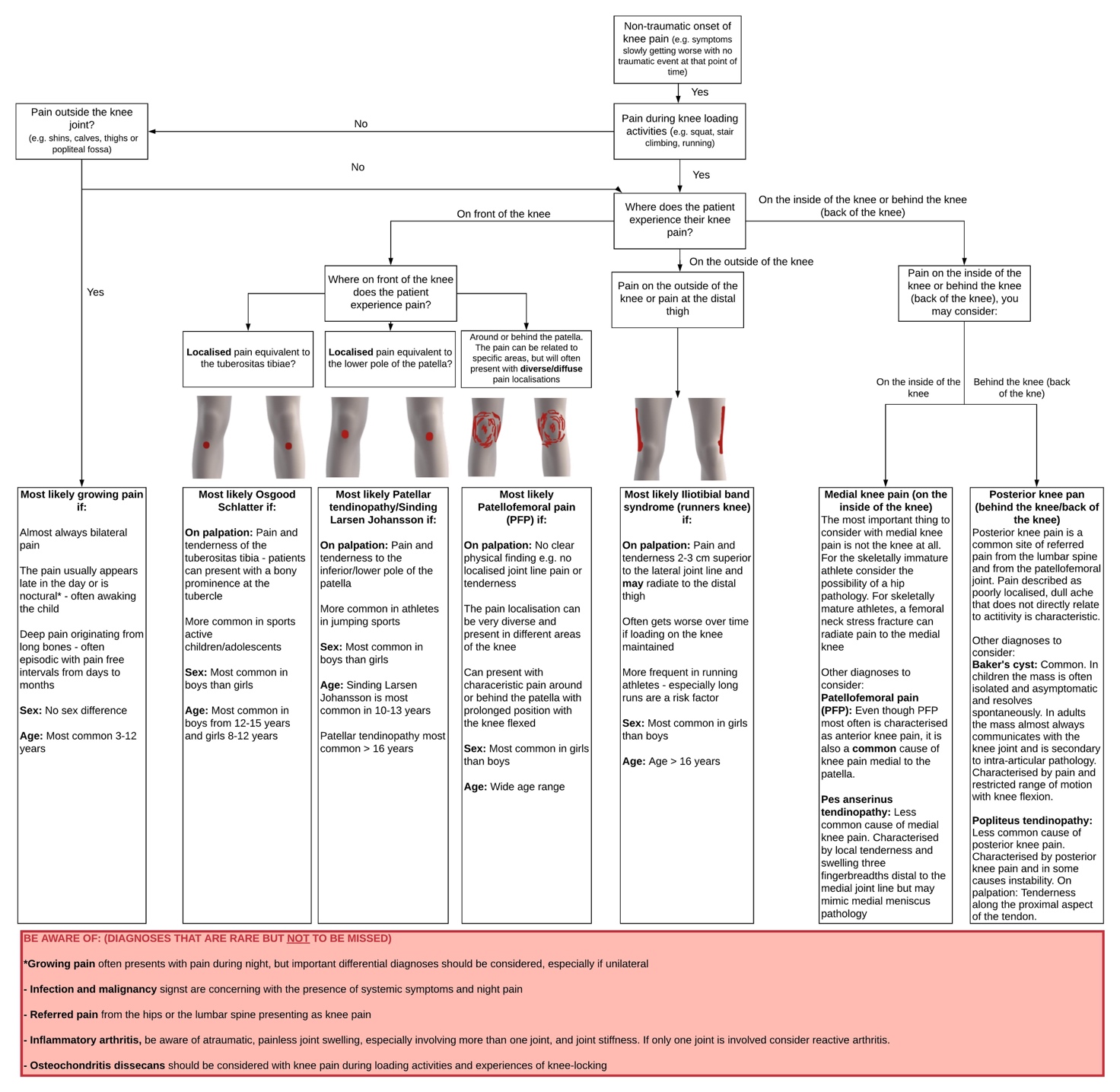
**

**Figure S18.**  Final version in English

References

1. Austermuehle PD: Common knee injuries in primary care. *Nurse Pract*. 2001*;26*(10):26, 32-47.

2. Boling M, Padua D, Marshall S, et al: Gender differences in the incidence and prevalence of patellofemoral pain syndrome. *Scand J Med Sci Sports*. 2010*;20*(5):725-730.

3. Circi E, Atalay Y, Beyzadeoglu T: Treatment of Osgood-Schlatter disease: review of the literature. *Musculoskelet Surg*. 2017*;101*(3):195-200.

4. Conrich I: ITBS - Review Article. *Journal of New Zealand & Pacific Studies*. 2019*;7*(1):89-94.

5. Cook C, Mabry L, Reiman MP, et al: Best tests/clinical findings for screening and diagnosis of patellofemoral pain syndrome: a systematic review. *Physiotherapy*. 2012*;98*(2):93-100.

6. Décary S, Frémont P, Pelletier B, et al: Validity of Combining History Elements and Physical Examination Tests to Diagnose Patellofemoral Pain. *Arch Phys Med Rehabil*. 2018*;99*(4):607-614.e1.

7. Dixit S, DiFiori JP, Burton M, et al: Management of patellofemoral pain syndrome. *Am Fam Physician*. 2007*;75*(2):194-202.

8. Finlayson C: Knee injuries in the young athlete. *Pediatr Ann*. 2014*;43*(12):282.

9. Gholve PA, Scher DM, Khakharia S, et al: Osgood Schlatter syndrome. *Curr Opin Pediatr*. 2007*;19*(1):44-50.

10. Kodali P, Islam A, Andrish J: Anterior Knee Pain in the Young Athlete: Diagnosis and Treatment. *Sports Medicine and Arthroscopy Review*. 2011*;19*(1):27-33.

11. Krause BL, Williams JP, Catterall A: Natural history of Osgood-Schlatter disease. *J Pediatr Orthop*. 1990*;10*(1):65-68.

12. Lack S, Neal B, De Oliveira Silva D, et al: How to manage patellofemoral pain - Understanding the multifactorial nature and treatment options. *Phys Ther Sport*. 2018*;32*:155-166.

13. McConnell J: Management of patellofemoral problems. *Man Ther*. 1996*;1*(2):60-66.

14. Medlar RC, Lyne ED: Sinding-Larsen-Johansson disease. Its etiology and natural history. *J Bone Joint Surg Am*. 1978*;60*(8):1113-1116.

15. Nijs J, Van Geel C, Van der auwera C, et al: Diagnostic value of five clinical tests in patellofemoral pain syndrome. *Man Ther*. 2006*;11*(1):69-77.

16. Parikh S, Shrivastava R: Evaluation of Children with Injuries Around the Knee. *Indian J Pediatr*. 2016*;83*(8):844-851.

17. Patel DR, Villalobos A: Evaluation and management of knee pain in young athletes: overuse injuries of the knee. *Translational pediatrics*. 2017*;6*(3):190-198.

18. Price JL: Patellofemoral syndrome: how to perform a basic knee evaluation. *JAAPA*. 2008*;21*(12):39-43.

19. Rathleff MS, Vicenzino B, Middelkoop M, et al: Patellofemoral Pain in Adolescence and Adulthood: Same Same, but Different? *Sports Med*. 2015*;45*(11):1489-1495.

20. Santana JA, Sherman Al: Jumpers Knee, in: *StatPearls.* Treasure Island (FL), StatPearls Publishing, 2019.

21. Slotkin S, Thome A, Ricketts C, et al: Anterior Knee Pain in Children and Adolescents: Overview and Management. *J Knee Surg*. 2018*;31*(5):392-398.

22. Taunton JE, Ryan MB, Clement DB, et al: A retrospective case-control analysis of 2002 running injuries. *Br J Sports Med*. 2002*;36*(2):95-101.

23. Uziel Y, Hashkes PJ: Growing pains in children. *Pediatric rheumatology online journal*. 2007*;5*(1):5.
