# Supplementary material for: Development and validation of the Sorting non-trauMatIc adoLescent knEe pain (SMILE) tool – a development and initial validation study
Source: Pediatr Rheumatol Online J. 2021 Jul 6;19:110. doi: 10.1186/s12969-021-00591-5 (PMC8259444; doi:10.1186/s12969-021-00591-5)
Supplement: Supplementary file 3 — Additional file 3. [file 12969_2021_591_MOESM3_ESM.docx]

# Additional file 3

| **Diagnoses without using the tool** | **Diagnoses using the tool** |
| --- | --- |
| OSD  Anterior knee pain  Overuse  Swimmers knee  Degeneration of the meniscus  Patellar tendinopathy  Injury to the ligament  Meniscus lesion  Collateral ligament lesion  PFP  Runners knee  MCL lesion  Bursa irritation  PCL | Patellar tendinopathy  Sinding Larsen Johansen  OSD  PFP Runners knee (iliotibial band syndrome) |
